# The Access Technology Program of the Indiana Clinical Translational Sciences Institute (CTSI): A model to facilitate access to cutting-edge technologies across a state

**DOI:** 10.1017/cts.2020.525

**Published:** 2020-08-19

**Authors:** Christie M. Orschell, Todd C. Skaar, Melanie E. DeFord, Joel Ybe, Julie Driscol, Christine Drury, Lilith Reeves, Monte S. Willis, Jill L. Reiter, Jenna York, Rob Orr, Jeanette N. McClintick, Thomas G. Sors, Joe Hunt, Kenneth Cornetta, Anantha Shekhar

**Affiliations:** 1 Department of Medicine, Indiana University School of Medicine, Indianapolis, IN, USA; 2 Indiana CTSI Access Technology Program, Indiana University School of Medicine, Indianapolis, IN, USA; 3 Notre Dame Research and Indiana CTSI Access Technology Program, University of Notre Dame, Notre Dame, IN, USA; 4 Office of the Vice Provost for Research and Indiana CTSI Access Technology Program, Indiana University School of Public Health, Bloomington, IN, USA; 5 Indiana CTSI Translational Research Development Program, Indiana University School of Medicine, Indianapolis, IN, USA; 6 Indiana CTSI Research Communications, Indiana University School of Medicine, Indianapolis, IN, USA; 7 Department of Medical and Molecular Genetics, Indiana University School of Medicine, Indianapolis, IN, USA; 8 Department of Pathology and Laboratory Medicine, Indiana University School of Medicine, Indianapolis, IN, USA; 9 Department of Biochemistry and Molecular Biology, Indiana University School of Medicine, Indianapolis, IN, USA; 10 Institute of Inflammation, Immunology and Infectious Disease and Indiana CTSI Access Technology Program, Purdue University, West Lafayette, IN, USA; 11 Indiana CTSI Tracking and Evaluation Program, Indiana University School of Medicine, Indianapolis, IN, USA; 12 Indiana CTSI, Indiana University School of Medicine, Indianapolis, IN, USA

**Keywords:** Translational research, technology, CTSI, core facilities, pilot funding

## Abstract

**Introduction:**

Access to cutting-edge technologies is essential for investigators to advance translational research. The Indiana Clinical and Translational Sciences Institute (CTSI) spans three major and preeminent universities, four large academic campuses across the state of Indiana, and is mandate to provide best practices to a whole state.

**Methods:**

To address the need to facilitate the availability of innovative technologies to its investigators, the Indiana CTSI implemented the Access Technology Program (ATP). The activities of the ATP, or any program of the Indiana CTSI, are challenged to connect technologies and investigators on the multiple Indiana CTSI campuses by the geographical distances between campuses (1–4 hr driving time).

**Results:**

Herein, we describe the initiatives developed by the ATP to increase the availability of state-of-the-art technologies to its investigators on all Indiana CTSI campuses, and the methods developed by the ATP to bridge the distance between campuses, technologies, and investigators for the advancement of clinical translational research.

**Conclusions:**

The methods and practices described in this publication may inform other approaches to enhance translational research, dissemination, and usage of innovative technologies by translational investigators, especially when distance or multi-campus cultural differences are factors to efficient application.

## Introduction

The Indiana Clinical and Translational Sciences Institute (CTSI) is uniquely conceived in that it serves the state’s research centers. The Indiana CTSI is a partnership among Indiana University, Purdue University, and the University of Notre Dame, combining the expertise of three large and preeminent research universities on four large research campuses. The Indiana CTSI is designed to bring together the state’s brightest minds to solve Indiana’s most pressing health challenges through research, education, and workforce development. The Indiana CTSI achieves its mission through its various programs, one of which is the Access Technology Program (ATP), the focus of this communication.

The overall goal of the ATP is to improve the impact and competitiveness of investigator research by integrating innovative technologies into research studies. The ATP encourages investigators to incorporate novel technologies into their research program and to assist those already using these technologies by improving the quality of core services. While the benefits of combining the expertise of three large research universities with four large research campuses across the state may seem obvious, the challenges presented are not easily overcome. In this paper, we share some insights we have gained to overcome these hurdles and consistently deliver access to cutting-edge technology across the state in order to conduct high-quality research for the benefit of Indiana’s citizens, the nation, and beyond. Challenges and solutions are presented in their respective sections.

### Mission of the ATP

**Fig. 1.**
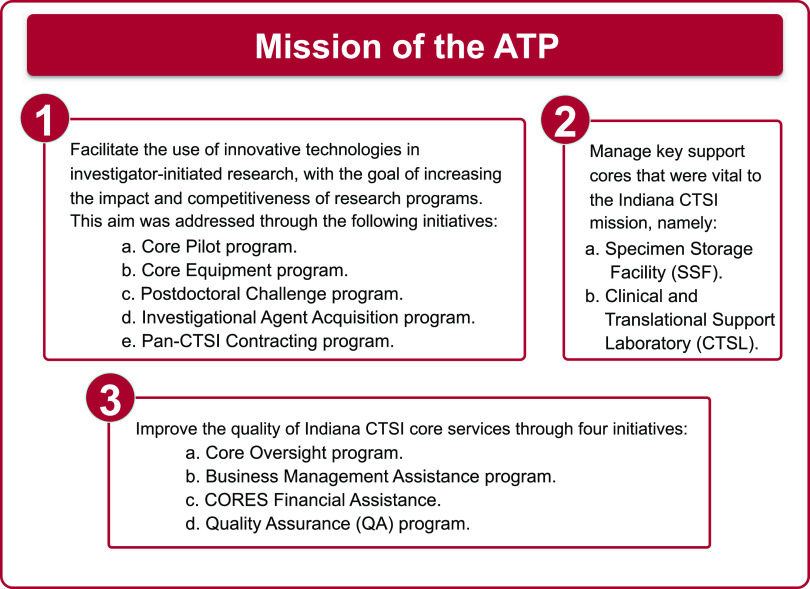
The three distinct programs of the Access Technology Program (ATP).

The mission of the ATP was formulated with three related but distinct programs, as shown in Fig. 1. Each of these programs and initiatives is discussed in detail in the following sections.

## Methods and Results

### Core Pilot Program

While novel technologies can enhance the significance of a research program, the Indiana CTSI recognized the challenge that investigators may lack the funds to generate sufficient preliminary data for grant applications. Responding to requests for additional data for submitted grants can also pose specialized research technology challenges. Our solution to these challenges was to develop the *Core Pilot program*, which is a competitive, investigator-initiated program that provides up to $10,000 for core services at any Indiana CTSI-Designated core. The program was initially started as a yearly competition, funding approximately 25 awards per year. In 2011, review sessions were increased to semi-annually at the suggestion of investigators. Selection of awards is based on a multi-institutional review committee that fosters interactions among the campuses. In addition to the scientific merit of the proposal, Core Pilot funding is awarded based on the following criteria: (i) obtaining critical preliminary data for grant applications; (ii) developing a new reagent or resource; (iii) testing a new idea or new line of research; or (iv) assisting in the development of intellectual property. The potential impact on future grant applications and intellectual property are key review criteria. Core Pilot grants are not intended to independently support the development of new core technologies; however, if that is part of the needs of investigators, development projects can be included in the Core Pilot applications.

**Fig. 2.**
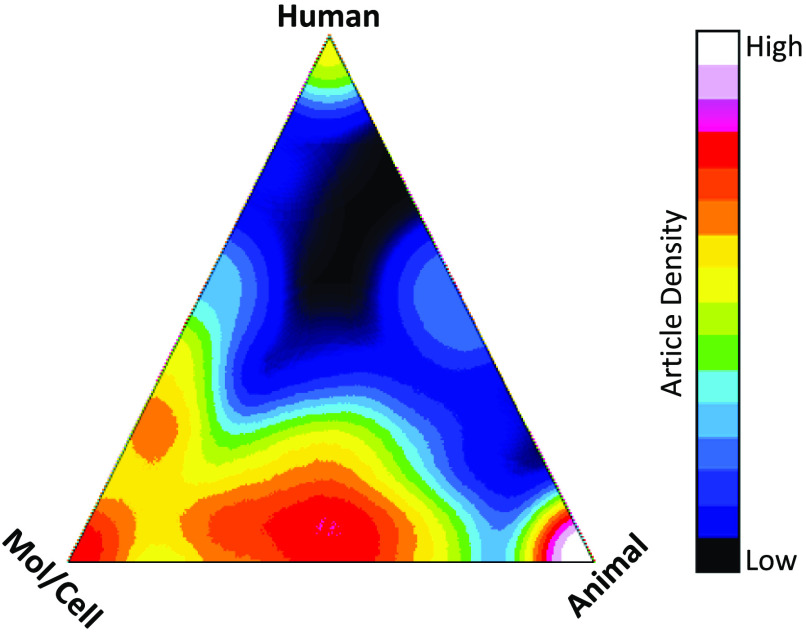
Analysis of the translational status of research reported. The graphic illustrates the degree of the translational status of papers published by Core Pilot awardees from 2009 through December 2019 in the Indiana CTSI. Data were obtained using the iCite tool (https://icite.od.nih.gov/analysis) accessed July 17, 2020.

The Core Pilot competition became highly popular and competitive, and the semi-annual review now evaluates between 60 and 90 applications per cycle. As part of the application, the applicant must include a signed letter by the core director(s) attesting to the work discussion, the ability of the core to perform the work, and the accuracy of the budget. This assures feasibility of the project and promotes interactions with the core experts and helps to raise awareness for the cores on each campus. The 2-page applications are reviewed in a National Institutes of Health (NIH)-style review by reviewers (two per application) recruited from across the three Indiana CTSI institutions based on the expertise needed for evaluating the applications. Awardees and non-awardees alike receive extensive feedback from the reviews that serves as a guide not only for any resubmissions, but also for the external funding submission following the project completion. Investigators receiving awards must provide progress reports, including information of publications and grant applications while conducting the project and for 5 years following the completion of the funding. Progress reports from the Core Pilots funded between 2009 and 2019 indicated that these projects contributed to the publication of 220 peer-reviewed publications and 225 funded grants totaling over $248 million. Table 1, which displays the breakdown of funding sources by application and award amount, shows that the majority of applications (58%) were to the NIH. Of the NIH awards, 44% (50 of 114 awards) represented nearly $67 million and were awarded to Assistant Professors. The 220 publications by Core Pilot awardees have been cited over 1900 times with a mean citation rate of 8.7 citations per publication and a median of 5 citations. The mean relative citation ratio (RCR) for publications with an RCR value was 1.24 indicating a higher than NIH average citation ratio. The research reported in these publications span the translational stages with average iCITE translation scores of human-based research (0.19), animal-based (0.32), and molecular/cellular (0.47) (Fig. 2).

**Table 1.** External grants data of core pilot awardees reported from 2009 through December 2019.

Source	Applications	Awards	Application amount	Award amount
Commercial	3	3	$1,829,999	$1,825,499
DoD	22	7	$37,222,709	$15,161,229
FDA	1	0	$600,000	$0
NIH	336	114	$476,854,558	$146,049,135
NSF	23	11	$15,044,922	$5,188,983
Private Foundations	161	75	$55,724,594	$27,084,143
State of Indiana	12	9	$2,125,768	$1,687,976
US Dept of Ed	1	0	$1,970,212	$0
US Dept of Energy	1	0	$750,646	$0
USAID	2	2	$200,000	$248,000
VA	14	6	$56,419,808	$51,400,000
Total	576	225	$648,743,216	$248,644,965

Funds awarded through the Core Pilot program can only be utilized in Indiana CTSI-Designated core facilities. To be an Indiana CTSI-Designated core requires the facility to meet benchmarks on services, user satisfaction, and transparency on cost and authorship issues (see *Core Oversight program*). Given the large number of core facilities within the 4 large university campuses (currently more than 60), the Core Pilot program facilitates Indiana CTSI support of cores through a mechanism that is investigator driven and vetted for scientific merit.

Peer review of applications also provides an important training ground for young investigators, addressing the education mission of the Indiana CTSI. In this initiative, first-time reviewers are paired with more senior reviewers to help provide balanced reviews. New reviewers frequently end up submitting applications the following round. The program also recently added an educational and service opportunity for outstanding postdoctoral fellows to serve as reviewers (see *Postdoctoral Challenge*).

One of the greatest challenges is recruiting enough reviewers with the appropriate expertise and no conflicts of interest to provide informed reviews and appropriate scores. Over 150 applications are received per year covering a wide range of translational research topics, which in turn, requires a wide variety of reviewer expertise. Reviewers have been selected by Indiana CTSI staff and the Core Pilot Chair and recruited for each cycle independently. We are now experimenting with a new approach by engaging the Indiana CTSI-Designated core directors to recommend faculty who could serve on a standing review committee, based on previous core usage and expertise, and have selected additional reviewers to fill gaps in expertise.

### Core Equipment Program

In addition to providing funding via the Core Pilot program, the CTSI manages a C*ore Equipment program* for the Indiana University (IU) School of Medicine. This program aims to further maximize the impact of our cores by providing funding to purchase equipment that will either establish a new scientific capability, expand access to more investigators, or replace existing outdated or unreliable instruments. Cores must be Indiana CTSI-Designated cores (see *Core Oversight program*) to be eligible for *Core Equipment Grants*. To date, the IU School of Medicine is the only campus that has allocated funds to this program.

The *Core Equipment program* provides an opportunity for the IU School of Medicine Indiana CTSI-Designated core directors to request support for the purchase of equipment and/or software. The goal is to enhance the research environment and contribute to the research mission of the school and the Indiana CTSI by expanding existing services, or providing for new technology and services. Each year, one cycle of funding is offered with a total institutional investment of $100,000. This mechanism is for equipment that costs between $5000 and $100,000. The cores can request up to the total amount offered each year. Equipment with higher costs are allowable if they can secure the funding to cover the balance of the item’s cost. This funding can be used for any combination of core applications. Since 2010, approximately 30 equipment requests have been awarded. Annual progress reports for Core Equipment awardees include a list of investigators who have used the new service/equipment, grant submissions and publications arising from data obtained from the new service/equipment, and a list of the publications that cited the core.

To enhance the research infrastructure on the Bloomington campus, the Indiana CTSI and the IU Bloomington Office of the Vice Provost for Research have coordinated to offer the Research Equipment Fund (REF). The aim of this funding mechanism is to support the purchase of critical equipment that increases the ability of researchers to secure extramural funding. To qualify, an applicant must be faculty on the IU Bloomington campus, but may include co-investigators at Indiana CTSI partner institutions. Up to $200,000 is available for two rounds per year (~$100,000 per round). To leverage departmental resources, there is a 50% match requirement from participating units.

The infrastructure enabled by the ATP program of the Indiana CTSI also allows for the creation of joint multi-university high-end cores and equipment to further facilitate access to cutting-edge technologies. As an example, six major life science entities – Purdue University, IU School of Medicine, IU Bloomington, the University of Illinois at Urbana-Champaign, Eli Lilly and Co., and the Indiana Biosciences Research Institute – strategically formed a consortium to deepen our regional strength in structural biology and have acquired a second Krios Cryo-Transmission Electron Microscope for Cryo-electron microscopy (Cryo-EM) applications. The new microscope will be located in the Cryo-EM Facility (a CTSI-Designated facility) on the Purdue University campus in West Lafayette, where there is a nucleus of expertise in structural biology. The facility is also an NIH-designated site for the Midwest Consortium for High-Resolution Cryoelectron Microscopy. Together with the CryoEM expertise and capabilities at Indiana University, the consortium has already been making biomedical breakthroughs from Alzheimer’s tau protein-aberrant structures to resolving the structure of the Zika virus. This partnership also acquired a Talos Artica single-particle CryoEM for the IU Bloomington campus to broaden access to the technology throughout the region. Cryo-EM technology is a “revolutionary technique for determining the 3D shape of proteins”, according to a recent editorial in Nature [1], which bombards flash-frozen solutions of biomolecules such as proteins with electrons to produce microscopic images of single molecules. Cryo-EM allows investigators to discover how proteins work and malfunction in diseases, in addition to lending information on how best to target these proteins with drugs in the future. By expanding the use of screening Cryo-EM outside of the Purdue hub, the Cryo-EM consortium optimizes the limited Krios Cryo-EM capacity with the continued high demand for this resource. In recent publications by IU faculty on Alzheimer’s tau proteins, screening in local EM Cores such as the IU School of Medicine has been essential to optimize the current central Purdue Cryo-EM resources. The consortium will continue to develop this pipeline throughout the state based on best practices for managing large Cryo-EM facilities [2]. The CryoEM consortium illustrates how the connections and collaborations resulting from the Indiana CTSI can foster the creation of a unique resource that can benefit many more investigators that otherwise would have been challenging on a local level.

### Postdoctoral Challenge

The Indiana CTSI *Postdoctoral Challenge* is a program intended to provide postdoctoral researchers with the opportunity to gain proposal development and peer review experience. Launched in October 2014, the program is designed to prepare postdoctoral investigators to be more effective translational researchers through the preparation of proposals that request they articulate a translational strategy for their project through a competitive granting mechanism. In the Postdoctoral Challenge, investigators prepare proposals for $5000 to use the services of Indiana CTSI-Designated core facilities (see *Core Oversight program*).

The Postdoctoral Challenge also addresses the Indiana CTSI’s education and workforce development mission by providing valuable training in grant writing and reviewing for postdoctoral investigators. In the Postdoctoral Challenge program, postdocs are also encouraged to participate as reviewers of these applications in review sessions with other peers, faculty, and Indiana CTSI staff from the participating Indiana CTSI institutions. The purpose is to give postdocs first-hand experience of a typical review session using NIH guidelines, scoring, and review criteria. Several months prior to the application submission deadline, postdocs have the opportunity to attend workshops focused on “Effective Grant Writing Techniques”, “Writing Translational Research Proposals”, and an “Overview of an NIH Review Session”. The workshops help postdoctoral investigators with proposal preparation and best practices, as well as covering the preparation of NIH biosketches, project budget, and reviewer guidelines. For most postdocs, this is their first opportunity to participate in a peer review session that provides them with an invaluable perspective of the review process and can help them improve the development of future proposals. As one participant noted: “*This review process was very helpful for me to understand the limitation of my own project proposal that I did not realize while writing my proposal*.” Exemplary postdocs that demonstrate an aptitude as reviewers are recommended to the Indiana CTSI administration to participate as reviewers for the Core Pilot program competition (see *Core Pilot Program* for details on this program) along with faculty reviewers. This provides the young investigators the opportunity to participate in a larger, NIH-type study section and meet new colleagues from across the Indiana CTSI campuses. It also helps them develop their grant writing expertise and learn to provide feedback on other applications.

### Investigational Agent Acquisition Program

The fourth approach to facilitating research by the ATP was through the *Investigational Agent Acquisition* (IAA) program, which assists investigators with the acquisition of investigational agents (drugs, devices, and biologics) and with regulatory submission. Members of the ATP along with the Regulatory Knowledge and Support (RKS) program and the Participant and Clinical Interactions Resources (PCIR) programs within the Indiana CTSI, combined efforts to identify suitable vendors, assist in negotiations, and even provide guidance if manufacturing was required. The IAA program has since been relocated within the Indiana CTSI to the Molecular Therapeutics program.

### Pan-Indiana CTSI Contracting Program

The fifth means by which the ATP facilitates the availability of research tools across the state of Indiana is through the *Pan-Indiana CTSI Contracting program*. This program approached multiple technology vendors for preferred rates by combining the buying power of the three Indiana CTSI institutions. Key areas for saving included bioinformatics tool licenses and large-volume sequencing outsourcing. The ATP’s most popular pan-licenses to date have been for bioinformatics tools, namely Thomson Reuters’ MetaCore and QIAGEN’s Ingenuity Pathway Analysis (IPA) licenses. Both of these products enable an analysis of large omics datasets but are expensive, with the license costs prohibitive for most investigators. Given that investigators use these tools intermittently, Pan-Indiana CTSI licenses have saved investigators thousands of dollars annually in licensing costs, and facilitated interpretation of experimental results and publication of data.

To implement this program, the Indiana CTSI negotiates a license for the products based on the expected number of users, then charges investigators for sub-licenses based on the number of subscribers. MetaCore was the first software with an Indiana CTSI-wide license and investigators were charged based on total annual usage. However, this model was cumbersome and not sustainable. With QIAGEN’s IPA, two smaller licenses were combined from Purdue and IU School of Medicine, which were both previously charging a flat rate for the annual license. This model for the CSTI-wide IPA license has worked so well, it has now been extended to MetaCore. In both cases, the companies involved allow the Indiana CTSI to handle the creation and deletion of user accounts including trial licenses. The usage of MetaCore has waned over the years, but the IPA usage has continually risen to the current level of approximately 70 active users per quarter from 15 original users. In addition, users from Notre Dame and Indiana University Bloomington have now joined the IPA pan-license.

For both MetaCore and IPA, the sharing of software has resulted in lower costs with more access. For example, each original IPA license allowed only one concurrent user, leading to frustration when the software was unavailable. Through the purchase of several multisite licenses, users from Indiana CTSI partner institutions now have greater access to IPA, increasing user satisfaction. The Indiana CTSI also arranges free annual training sessions at three different locations to introduce new users to the IPA software. Training occurs about 6 weeks before the new licensing period, allowing potential users the opportunity to evaluate the software more thoroughly before committing to the purchase of a 1-year sublicense. It also allows labs with existing licenses to train new employees. The cost and frustration resulting from limited licenses has been eased for investigators through implementation of the Indiana CTSI-wide licenses.

### Specimen Storage Facility

The ATP also identified two key facilities that were of size or importance to warrant central management. The Indiana CTSI *Specimen Storage Facility* (SSF, https://indianactsi.org/researchers/services-tools/biospecimen-services/biomanagement/) currently occupies 5858 net square feet of space at the IU School of Medicine Indianapolis campus. The initial facility included a 1702 square foot room specifically designed with dedicated heating, ventilation, and air conditioning (HVAC) and a comprehensive alarm system to support 54 ultra-low-temperature freezers and a second 978 square foot liquid nitrogen room with an initial cryogenic (LN2) freezer capacity of 295,100 vials. This high usage facility houses a number of national and local biobanks allowing sample ownership and management to remain with the biobank managers.

Today, the SSF continues to assist clinical investigators across multiple departments and throughout the Indiana CTSI partner institutions and includes investigators with large efforts such as the Indiana Biobank, IU Simon Cancer Center Tissue Bank, Komen Tissue Bank, Michael J. Fox Foundation, the NIH National Cell Repository for Alzheimer’s Disease, the National Heart, Lung, and Blood Institute (NHLBI) National Gene Vector Biorepository, Vector Production Facility, and the Cell and Gene Therapy Manufacturing Group. Most recently, the facility is banking COVID-19 research samples for genomics and cytokine analyses via a joint venture between the Indiana CTSI and the IU Grand Challenge Precision Health Initiative, which will make data available to any investigator conducting COVID-19 research. The facility is Standard Operating Procedure (SOP)-driven with an active quality oversight program in place that adheres to the International Society for Biological and Environmental Repositories (ISBER) [3] comprehensive list of best practice guidelines for biobanks.

Although investigators were initially hesitant to pay for services that prior to the formation of the SSF were held within Principle Investigator (PI) labs, once they recognized the value the SSF was providing, the program became popular and continues to grow and expand today. The SSF has addressed the challenges of increased demand with creative solutions. Two expansion projects added 2300 square feet to the facility, increasing capacity to 110 ultra-low-temperature freezers and 17 liquid nitrogen freezers. Efforts are ongoing to improve inventory management strategies, develop standard practices and services for purchasing, and to implement a change of the SSF model to allow monitoring and maintaining freezers in all labs on the IU School of Medicine Indianapolis campus. Enhancements over the years have included a secondary/redundant alarm system that added continuous temperature monitoring and increased sample safety, continuous improvements to operating procedures to meet evolving regulations and challenges, as well as a comprehensive freezer maintenance program, all with the goal to ensure the facility is utilized to its maximum potential. In addition, a Six Sigma process was recently initiated with the goal to optimize operations of the IU Bio-Repositories by providing oversight for the use, management, and integrity of biobank samples and data across their lifecycle, with a focus on supporting the IU School of Medicine mission and goals, providing value, minimizing risk, and complying with applicable regulations.

### Clinical and Translational Support Laboratory (CTSL)

The ATP also manages the *Clinical and Translational Support Laboratory* (CTSL), located at the IU School of Medicine Indianapolis campus in the Clinical Research Center of the Riley Hospital for Children at IU Health. The CTSL processes samples for many clinical trials conducted in the Indiana CTSI Clinical Research Center and throughout the campus. The CTSL developed a quality program with SOPs for laboratory management, equipment maintenance, sample processing, storage, and shipping in compliance with appropriate regulatory guidance. The ATP began processing samples in 2010 and processed 4755 samples that year and 6806 in 2011. The CTSL has grown and evolved over the past 10 years. While the original focus was to support clinical trials that were conducted in the Clinical Research Center located in IU Hospital, support now is extended to nearly every department within the IU School of Medicine. In 2018, the CTSL provided support to 188 clinical trials that included industry and PI-initiated research conducted in IU Health University Hospital, Riley Hospital for Children, Eskenazi Health, Veteran Health Indiana, and IU Health Methodist Hospital, and Purdue University in addition to collaborating facilities and other CTSI institutions around the nation.

Tens of thousands of individual specimens are accessioned, processed, and shipped by the CTSL each year. Individualized support, such as specimen collection/processing guidance, specimen label creation, assistance with monitor audits, site initiation visits, and documentation requests, is provided by the CTSL so that research coordinators and PIs can concentrate on other priorities. Utilization of standardized practices, while remaining flexible enough to meet protocol-specific requirements, enables the CTSL to provide quality and consistency for every protocol that passes through the lab.

As protocol requirements evolved over time, the CTSL added services to meet the growing needs. Shipping services were added in 2014, peripheral blood mononuclear cell (PBMC) processing was added in 2016, and DNA extraction from whole blood was added as a new service in early 2019. Future projects include implementing a process for electronic data capture of critical processing and storage information, lab manual templates for Investigator-Initiated Trials, and enhanced quality control.

### Core Oversight Program

Similar to other initiatives around the country [4–8], it was important that the Indiana CTSI develop a system to promote best practices and foster quality of core services for Indiana CTSI investigators. Given that the cores were geographically located on four campuses and administratively managed by individual school or departmental units, the challenge of implementing such a system was recognized. While managing the large number of cores in the three Indiana CTSI institutions was beyond the scope of the Indiana CTSI, the CTSI could play an important role in advancing quality of services, thereby bringing value to the core and the faculty that utilize core services. This was accomplished in the ATP through development of the *Core Oversight program*, a voluntary accreditation process whereby cores on any Indiana CTSI campus that meet rigorous quality standards for scientific quality, pricing, operations, advisory committees, policies governing publication, payment and dispute resolution, user satisfaction, and reporting are granted “Indiana CTSI-Designated core” status. This designation lets researchers know that this core has policies, rates, an advisory committee, and survey results that have been reviewed by a committee representing all the Indiana CTSI partners, and gives researchers confidence that the operations and processes of the core are assessed and appropriate. The number of Indiana CTSI-Designated cores has grown by 30% since implementation of the program in 2010 (46 cores in 2010) to 60 Indiana CTSI-Designated cores in 2019. As mentioned earlier in this communication, Indiana CTSI-Designated cores are eligible for the Core Pilot and Core Equipment programs.

Since the cores are collectively reviewed by ATP liaisons (described in the “*Staying connected*” section below) from all Indiana CTSI campuses, the *Core Oversight program* also provides the ATP with information on capabilities of the cores on the various campuses, which can be passed onto investigators at their home institutions. The core oversight and approval process also ensures uniformity of standards across multiple campuses. The core website (which is undergoing reconstruction for improved utility) allows researchers to choose from facilities across the campuses, giving them more options with lower costs rather than paying higher prices for services outside their institution. User surveys are examined for user satisfaction and any issues addressed as needed.

### Business Management Assistance Program (BMAP)

The BMAP was developed at the suggestion of core directors to assist in implementing efficient business practices. The ATP partnered with the IU Kelley School of Business who pairs three to five MBA students with an Indiana CTSI core facility. Core selection is based on a yearly competition and the BMAP has assisted up to 8 cores per year [9,10]. Priority is given to proposals that will: (1) lead to improvements in organizational efficiency, speed of service, and/or quality; and (2) can potentially be extrapolated and benefit other cores, resources, programs, or units. The proposal must request and define a need for assistance in one or at most two of these areas: Project Management, Marketing, Financial management, and Resource Efficiency Management. Core directors reported that their experience in the BMAP led to improvements in core visibility and utilization, website development, marketing materials, training programs for core personnel, analysis of user satisfaction surveys, staff engagement, and an overall more favorable core environment.

### Financial Assistance

To assist with financial management, the IU School of Medicine and the University of Notre Dame originally purchased the Vanderbilt University Core Ordering & Reporting Enterprise System (CORES) management program to manage ordering, invoicing, and track core services. The ATP was charged with assisting the various core facilities in implementing the software program. Agilent Technologies Inc. has recently acquired Vanderbilt CORES, which is being phased out and replaced by iLab Operations software. Purdue University uses iLab and the other three Indiana CTSI campuses are transitioning to iLab as well, which will provide new opportunities for cross-use of core facilities when all campuses are on the same system. Agilent will install the iLab system and provide web-based training for users.

### ATP Quality Assurance (QA) Program

The ATP initiated an SOP-driven quality management program to monitor services, identify deficiencies, and implement corrective action when needed for the SSF and CTSL. This QA program reviews SOP-driven activities and conducts periodic QA audits. The ATP is also responsible for responding to findings during external audits. The ATP assigns tasks to the QA specialist, but to allow independence, the QA specialist is directed to report significant findings to the IU Office of Compliance.

## Staying Connected

Adenosine triphosphate (ATP) is a complex organic chemical that provides energy to drive many processes in living cells, e.g., muscle contraction, nerve impulse propagation, and chemical synthesis. Found in all forms of life, ATP is often referred to as the “molecular unit of currency” of intracellular energy transfer. It is the high-energy molecule that stores the energy we need to do just about everything we do. Just as biologists view ATP as vital and being key to all we do, so is the ATP of the Indiana CTSI. Core facilities and other research resources play a large part in supporting and seeding research across the Indiana CTSI research partners.

To maximize usage of the Indiana CTSI ATP, the program leaders have developed innovative strategies to connect minds and talent on all four campuses. Key to this connectivity is the *ATP liaison*, the ATP representative from each campus. There are currently six liaisons and one program coordinator for the ATP. The liaisons are contact points for both investigators and Indiana CTSI-Designated core directors, and also serve on the Indiana CTSI Project Development Teams with the specific charge to identify projects which would be enhanced by incorporating new technologies, further fostering core use. Many of the liaisons have been in the role since the inception of the grant (2008), allowing continuity that has fostered close relationships among the campus representatives, which in turn fosters support of the needs of the grant and each other’s research community. This long-standing partnership has fostered a rapport among the liaisons. They reach out to each other for all things core-related, big or small. Communication is key. This group works very well together, all for the benefit of the Indiana CTSI. The ATP liaisons meet monthly via teleconference to discuss ongoing and new initiatives, brainstorm new ways to support access to technology, gain approval from the CTSI Executive committee when necessary, and then work with their home campuses to put ideas into action.

The many initiatives of the ATP also help to connect campuses and facilitate interactions. The ATP maintains a web-based *Help Desk* to assist investigators in need of core services, identifying options within the Indiana CTSI, other CTSIs, and other academic institutions or commercial vendors. The Indiana CTSI website provides information on 60 core programs throughout the Indiana CTSI. In the first full year of use, the Indiana CTSI website (www.indianactsi.org) was accessed 1226 times by 524 unique users. In 2018, Services Cores pages on the Indiana CTSI website were accessed 6780 times. The *Postdoctoral Challenge program* has training programs on all campuses, and allows the interaction of postdocs from all of the campuses in the joint review process. The *Indiana CTSI-Designated Core program* is another example of the liaisons working together through a joint review of the cores and discussions at monthly meetings, allowing the liaisons to gain knowledge of technologies available on partner campuses that can be passed onto investigators at their home institutions. The *Core Pilot program* fosters collaboration across campuses through joint reviews and access to previously unattainable technologies in the Indiana CTSI-Designated cores. This competition allows researchers access to first-class facilities and vital instrumentation on partner campuses that may not otherwise be available on their local campuses. This access further facilitates collaboration across partner institutions. This can be especially helpful for the regional campuses of the IU School of Medicine. As an example, several Core Pilot awards have been awarded to the IU School of Medicine South Bend faculty members for use of Notre Dame cores.

**Fig. 3.**
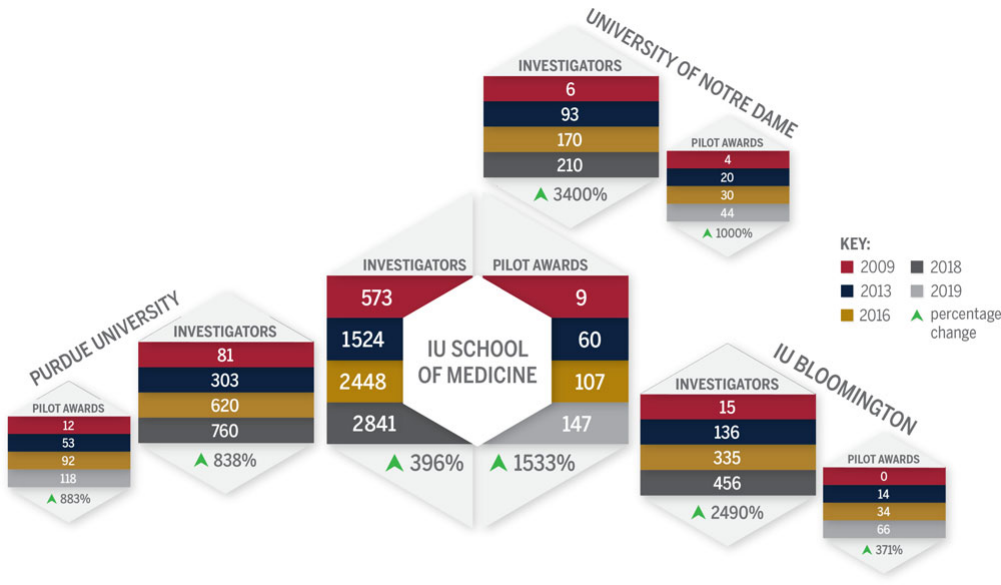
Cross-campus connections graph. This graph displays data for the number of investigators and percentage change of investigators from years 2009 through 2019 on each of the four regional campuses, and the number of pilot awards and percentage change of pilot awards on each campus for the same time frame.

The impact that the Core Pilot program has had on investigator productivity cannot be underestimated. Fig. 3 shows the number of CTSI investigators on each of the four regional campuses by year, and the number of Core Pilot awards for each campus per year. Evident in this figure is the incredible growth in both the number of investigators and percent increase in the number of Core Pilot awards.

In summary, this paper describes our experience in developing an effective program to deliver access to new and innovative technologies to translational research investigators spanning three institutions and four major campuses in the state of Indiana. Pilot grant programs incentivize use of cores and support acquisition of new equipment, leading to more robust cores with a broader clientele base. Pan-Indiana CTSI contracts provide investigators access to informatics programs at a fraction of the cost of single-user contracts. Support of key cores deemed essential to the success of clinical trials (SSF and CTSL) has resulted in growth and increased utilization of these cores, contributing to their vitality. Finally, applying quality oversight measures to the cores has strengthened them, increased user confidence in the cores, and aided core directors with operational procedures. These model systems developed by the ATP of the Indiana CTSI may be useful to other CTSIs or institutional administrative personnel in the development of more effective programs to advance translational research and enhance the interaction and collaborative activities of researchers spread across multiple campuses. The majority of these systems put in place 12 years ago with the initiation of the Indiana CTSI have not only stood the test of time but have grown to address increasing demand and innovations in research technology to support CTR. We believe this provides a potential model to other academic institutions and CTSAs with geographically dispersed partners to maximize access to and utilization of novel technologies.
